# Rapid assessment of avoidable blindness and diabetic retinopathy in individuals aged 50 years or older in Costa Rica

**DOI:** 10.1371/journal.pone.0212660

**Published:** 2019-02-21

**Authors:** Róger Iván Acevedo Castellón, Esteban Carranza Vargas, Ritzi Elena Cortés Chavarría, Gabriel Andrés Rodríguez Vargas

**Affiliations:** Ophthalmology Service, Surgery Department, México Hospital, Caja Costarricense de Seguro Social, Uruca, San José, Costa Rica; Help Me See, MEXICO

## Abstract

In the present study, we examined the causes and the prevalence of avoidable blindness and visual impairment, as well as the prevalence of diabetic retinopathy in individuals aged ≥50 years in Costa Rica, in order to provide baseline data for the initial planning and monitoring of ongoing blindness intervention programs. The assessment was based on the standardized methodology of the Rapid Assessment of Avoidable Blindness and Diabetic Retinopathy, a population-based survey for blindness and visual impairment. From 3,255 eligible subjects, 76.6% were examined. The adjusted prevalence of bilateral blindness [presenting visual acuity (VA) in the better eye of less than 3/60] was 1.7% (95% confidence interval, 1.2%–2.2%), with avoidable causes (treatable and preventable) accounting for 68.8% of cases. The main causes of blindness were cataract (52.1%), glaucoma (6.3%), and diabetic retinopathy (6.3%); these data were similar to those for other neighboring countries. Cataract surgical coverage (CSC) in the survey area was estimated as 88.9% for individuals with blindness (VA, <3/60), 76.6% for those with a VA of <6/60, and 60.3% for those with a VA of <6/18. The most common barriers against cataract surgery in individuals with a best-corrected VA of ≤6/60 included “need not felt” (48.8%) and “fear” (14.6%). Among individuals with a past history of cataract surgery, only 57.1% showed a “good” or “very good” outcome (VA, ≥6/18). Finally, 23.5% individuals with known or newly diagnosed diabetes showed retinopathy and/or maculopathy, with 6.2% exhibiting sight-threatening diabetic retinopathy (proliferative retinopathy, referable maculopathy, or both). Our findings indicate the need to overcome barriers against surgery for cataract, which is the main cause of avoidable blindness, to increase CSC, and to improve surgical outcomes. Moreover, improved methods for diabetic retinopathy screening can ensure prompt identification of patients with a risk of blindness. Glaucoma screening is also necessary for areas with a high prevalence.

## Introduction

The estimated worldwide prevalence of visual impairment [visual acuity (VA), <6/18] is 4.25%, with 14% of individuals considered to exhibit blindness (VA, <3/60). However, the blindness is curable or preventable in approximately 80% of cases [1]. Consequently, in May 2013, the 66th World Health Assembly endorsed Resolution WHA66.4, titled “Universal Eye Health: A Global Action Plan 2014–2019.” According to this resolution, the prevalence of avoidable blindness in 2019 should be 25% lower than the 2010 baseline established by the World Health Organization in each Member State. This target can be achieved if a strategy to strengthen the provision of ophthalmic services is structured on the basis of recent assessments of the specific situation within each country. Therefore, the main objective of this plan is to address the need to generate scientific data on the magnitude and causes of visual impairment and the availability of ophthalmic services, and to use these data to monitor progress, define priorities, and encourage greater political and financial commitments concerning ocular health in each Member State [2].

Costa Rica is a country under a democratic regime. It is politically and economically stable, with an estimated population of 4.9 million at the end of 2017. From 2014, a year before the completion of the survey, to 2017, the total national health care expenditure was equivalent to 9.31% of the country´s gross domestic product. The State shows strong participation in the provision of health services through the Costa Rican Social Security Fund (“Caja Costarricense de Seguro Social”), a public institution in existence for almost 75 years. The health insurance system registers approximately 1,600,433 taxpayers and offers coverage to approximately 94.4% of the total population. Approximately 2,000 health facilities provide the health services required by this population, with different types of hospitals (specialized, 6; national, 3; regional, 7; and peripheral, 13) and a diverse range of less complex centers. The provision of specialized services in ophthalmology is concentrated at the hospital level.

Costa Rica has 183 active ophthalmologists; the number of ophthalmologists per million individuals is low and approximately half of the average for Latin America [3]. Moreover, there is a disparity between the growth rate for ophthalmologists and that for individuals aged ≥60 years [4].

In the present study, we estimated the prevalences and causes of avoidable blindness and visual impairment along with the prevalence of diabetic retinopathy in individuals aged ≥50 years in Costa Rica, in order to provide baseline data for the initial planning and monitoring of ongoing blindness intervention programs.

## Materials and methods

The Rapid Assessment of Avoidable Blindness and Diabetic Retinopathy (RAAB+DR) survey was developed in 2005 by the International Centre for Eye Health, London. It is a population-based survey with a standardized methodology that evaluates the burden of visual impairment and ocular health care services among individuals aged ≥50 years, who exhibit a greater disease burden in terms of avoidable blindness and visual impairment [5]. Approximately 1,051,708 individuals aged ≥50 years and living in Costa Rica were considered eligible for the study according to estimations of the National Statistical and Census Institute (INEC) for the second half of the year 2015.

This study was conducted in Costa Rica between September and October 2015. The research protocol adhered to the guidelines of the Declaration of Helsinki and was approved by the Institutional Ethic-Scientific Committee of Caja Costarricense de Seguro Social (CCSS) on September 14, 2015 (protocol number: R015-SABI-00073). Written informed consent was obtained from all participants prior to examination.

The manual and software for RAAB+DR are distributed by the International Center for Eye Health, London [6]. RAAB+DR uses multistage cluster sampling with a cluster size of 35 when the diabetic retinopathy module is included. Clusters are selected from a sampling frame by using systematic sampling with a probability proportional to the size of the population.

The sample size was calculated using Table 1 parameters.

**Table 1:** Parameters used to calculate sample size for the survey in Costa Rica using RAAB Software.

	*Population Size*	*Estimated Prevalence*	*Worst Acceptable*	*Noncompliance*	*Design Effect*
*Blindness*	1,051,708	2.00%	1.40%	10%	1.4
*Diabetic Retinopathy*	1,051,708	3.71%	2.90%	10%	1.4

These data are based on previous RAAB surveys in Latin America [7] and surveys for assessing the prevalence of diabetes in Costa Rica [8]. According to the RAAB+DR methodology, the estimated prevalence of diabetic retinopathy was 25% of the general prevalence of diabetes mellitus. The “worst acceptable” prevalences of blindness (70% of the estimated prevalence) and diabetic retinopathy (78% of the estimated prevalence) were adjusted in order to obtain a matching sample size. This resulted in a sample of 3,255 individuals aged ≥50 years, with 93 clusters of 35 individuals for each condition.

As described above, 93 clusters were selected from 1,060 clusters representing the entire population aged ≥50 years; this yielded a sample of 3,255 individuals. Households were selected by the compact segment methodology. The population unit was subdivided into segments, with each segment comprising a large enough population to provide the required number of individuals for the cluster. Then, one segment was randomly selected, and all households in that segment were visited. All individuals aged ≥50 years who had lived in the selected household for more than 6 months in the previous year were invited to participate, and those who provided written informed consent were included in the study and examined. When subjects were absent at the time of our visit, we scheduled a second visit on an appointment basis. Survey forms were prepared for subjects who were absent for a long period or refused to participate, and relatives or neighbors were asked to indicate the age, sex, and vision status (presence or absence of blindness). Eligible individuals who were absent or refused to participate were not replaced in order to prevent bias. Nonresponders were included in the number of eligible individuals, although their data were excluded from our analyses.

Before initiation of the fieldwork, four examiner teams were formed to conduct the survey. They underwent a formal training program conducted by a certified RAAB trainer, where they learnt how to use standard procedures for the identification of eligible subjects, assessment of VA, examination of the lens and retina, establishment of the main cause of blindness or visual impairment, and recording of data. Interobserver variability measured at the end of the training process for each of these parameters was found to be good or better (kappa coefficient, >0.6).

The eligible subjects were examined and interviewed in their homes by senior ophthalmic residents between Monday and Friday from 9 am to 7 pm. VA was measured with available distance correction using “E” Snellen optotypes of size 12 (VA, 6/12), 18 (VA, 6/18), and 60 (VA, 6/60) placed at 6 m. Size 60 was also used at 3- and 1-m distances for the measurement of VAs of 3/60 and 1/60. VA measurements were performed in daylight, one eye at a time. The pinhole VA was used as a surrogate for best-corrected vision. First, the E chart was shown from a close distance, the procedure was explained, and the examinee was instructed to point at the open ends of the letters. We started with the size 60 optotype placed at 6 m in order to check whether the patient understood the procedure. If the optotype was correctly visualized, smaller sizes were shown. In cases of inappropriate visualization, we started with bigger sizes and moved to smaller sizes until the patient failed to identify the optotype.

For lens status evaluation, all eligible subjects were taken inside the house and examined with a portable slit lamp (Reichert 15090 PSL) in semi-dark conditions. At this point, examiners determined the principal disorder associated with visual impairment and acquired details about cataract surgery from those who had undergone the procedure. Patients who had not undergone cataract surgery were asked to provide reasons.

For the determination of the glycemic status, all survey participants, regardless of a previous diagnosis of diabetes, underwent a random blood glucose (RBG) test using a portable glucometer (Accu-Chek Performa) based on the finger prick method. All known diabetics, defined as individuals who knew about their diabetes and were advised to use medication, and newly diagnosed diabetics, defined as individuals with an RBG level of ≥200 mg/dl who were unaware of their condition, underwent tests for diabetic retinopathy in both eyes. The examination was performed inside the house with the subject comfortably seated. The pupil was dilated with a short-acting mydriatic (tropicamide 0.5%–phenylephrine 5%), and the retina was examined with a portable indirect ophthalmoscope (All Pupil II Keeler). The Scottish Diabetic Retinopathy Grading Scheme, which is similar to the International Council of Ophthalmology grading system for diabetic retinopathy and diabetic macular edema, was used. Individuals with diabetes were questioned about their diabetes (age of onset, current treatment, and last eye examination date) using a standardized method, and answers were assigned to the corresponding category.

For data collection, data analysis, and report generation, a standardized survey form was used and entered into a specially developed database program (standardized in RAAB+DR studies). The RAAB package for Windows is programmed in Visual FoxPro version 9.0, and reports are generated through Crystal Reports 10. Both programs are runtime versions, and the user is authorized to use these free of charge.

## Results

### Blindness and visual impairment

In total, 2,493 of the 3,255 eligible subjects were examined (76.6%). The response rate was lower for individuals aged 50–60 years, particularly men (Table 2), who were generally not home at the time of the first and second visits because of their jobs.

**Table 2:** Proportions of men and women living in the survey area in Costa Rica and those who were actually examined (2015).

	Men				Women			
	*Examined*		*Survey Area*		*Examined*		*Survey Area*	
	**n**	*%*	N	*%*	**n**	*%*	N	*%*
50–59 years	**317**	*34*.*3%*	**254,240**	*49*.*8%*	**676**	*43*.*1%*	**261,033**	*48*.*3%*
60–69 years	**313**	*33*.*9%*	**149,616**	*29*.*3%*	**512**	*32*.*6%*	**155,567**	*28*.*8%*
70–79 years	**194**	*21*.*0%*	**72,698**	*14*.*2%*	**252**	*16*.*1%*	**79,634**	*14*.*7%*
≥80 years	**99**	*10*.*7%*	**34,266**	*6*.*7%*	**130**	*8*.*3%*	**44,654**	*8*.*3%*
**Total**	**923**	** **	**510,820**	** **	**1,570**	** **	**540,888**	

*Source: National Institute of Statistics and Censuses (INEC) of Costa Rica

The age- and sex-adjusted prevalence of blindness was 1.7% [48/2493; 95% confidence interval (CI): 1.2%–2.2%; Table 3], and the blindness was avoidable (treatable and preventable) in 68.8% of cases.

**Table 3:** Age- and sex-adjusted prevalences of different levels of visual impairment in individuals aged ≥50 years in Costa Rica (2015).

	Men		Women		Total	
Level of Visual Impairment	n	% (95% CI)	n	% (95% CI)	N	% (95% CI)
Blind (PVA, <6/120)	8,137	1.6 (0.7–2.5)	9,958	1.8 (1.3–2.4)	18,096	1.7 (1.2–2.2)
SVI (PVA, <6/60 and ≥6/120)	9,416	1.8 (0.7–3.0)	8,695	1.6 (0.5–2.7)	18,111	1.7 (0.9–2.6)
MVI (PVA, <6/18 and ≥6/60)	42,205	8.3 (5.8–10.8)	49,477	9.1 (7.5–10.8)	91,683	8.7 (7.1–10.3)
EVI (PVA, <6/12 and ≥6/18)	64,049	12.5 (9.9–15.2)	79,831	14.8 (11.9–17.6)	143,880	13.7 (11.1–16.2)

SVI: severe visual impairment, MVI: moderate visual impairment, EVI: early visual impairment; CI: confidence interval, PVA: presenting visual acuity (with available correction).

The prevalence of blindness without adjustment for age tended to increase exponentially with age; it was 0.7% (95% CI: 0.2%–1.2%) among individuals aged 50–60 years and as high as 7.4% (95% CI: 4.1%–10.7%) among individuals aged ≥80 years (Table 4).

**Table 4:** Prevalence of blindness (BCVA, <6/120) according to the age group and sex among individuals aged ≥50 years in Costa Rica (2015).

	Men		Women		Total	
Age group	n	% (95% CI)	n	% (95% CI)	N	% (95% CI)
50–59 years	3	1.0 (0.0–2.0)	4	0.6 (0.0–1.2)	7	0.7 (0.2–1.2)
60–69 years	3	1.0 (0.0–2.0)	6	1.2 (0.3–2.1)	9	1.1 (0.3–1.8)
70–79 years	5	2.6 (0.4–4.8)	10	4.0 (1.7–6.3)	15	3.4 (1.7–5.0)
80+ years	7	7.1 (2.3–11.8)	10	7.7 (3.2–12.2)	17	7.4 (4.1–10.7)
All age groups	18	2.0 (1.1–2.8)	30	1.9 (1.3–2.5)	48	1.9 (1.4–2.4)

BCVA: best-corrected visual acuity, CI: confidence interval

Untreated cataract (considered potentially curable through surgical treatment) was the main cause of blindness, accounting for 52.1% of cases (Table 5), followed by other posterior segment diseases (27.1%), glaucoma (6.3%), and diabetic retinopathy (6.3%). The two lattermost conditions, along with complications of cataract surgery (2.1%), are preventable causes.

**Table 5:** Distribution of subjects according to the main cause of different levels of visual impairment in individuals aged ≥50 years in Costa Rica (2015).

	Blindness		SVI		MVI		EVI	
	n	%	n	%	n	%	n	%
***1*. *Refractive error***	0	0.0%	0	0.0%	52	21.0%	202	54.7%
***2*. *Aphakia*, *uncorrected***	0	0.0%	0	0.0%	1	0.4%	0	0.0%
***3*. *Untreated cataract***	25	52.1%	34	66.7%	148	59.7%	117	31.7%
***4*. *Cataract surgery complications***	1	2.1%	3	5.9%	9	3.6%	8	2.2%
***5*. *Corneal opacity due to trachoma***	0	0.0%	0	0.0%	0	0.0%	1	0.3%
***6*. *Other corneal opacities***	1	2.1%	1	2.0%	0	0.0%	0	0.0%
***7*. *Ptisis bulbi***	0	0.0%	0	0.0%	0	0.0%	0	0.0%
***8*. *Onchocerciasis***	0	0.0%	0	0.0%	0	0.0%	0	0.0%
***9*. *Glaucoma***	3	6.3%	5	9.8%	9	3.6%	10	2.7%
***10*. *Diabetic retinopathy***	3	6.3%	1	2.0%	7	2.8%	2	0.5%
***11*. *ARMD***	0	0.0%	1	2.0%	6	2.4%	12	3.3%
***12*. *Other posterior segment diseases***	13	27.1%	5	9.8%	14	5.6%	14	3.8%
***13*. *Other anomalies of the globe or CNS***	2	4.2%	1	2.0%	2	0.8%	3	0.8%
***Total***	***48***	***100*.*0%***	***51***	***100*.*0%***	***248***	***100*.*0%***	***369***	***100*.*0%***

SVI: severe visual impairment, MVI: moderate visual impairment, EVI: early visual impairment, ARMD: age-related macular degeneration, CNS: central nervous system

### Cataract as the main cause of avoidable blindness

Untreated cataract accounted for blindness in 52.1% individuals, and it is considered the main cause of moderate (59.7%) or severe (66.7%) visual impairment in Costa Rica.

The estimated cataract surgical coverage (CSC) increased with a decrease in VA (Table 6), being 88.9%, 76.6%, and 60.3% for individuals with blindness, a VA of <6/60, and a VA of <6/18, respectively. This could be attributed to prioritization of individuals with more severe vision loss by ophthalmic care services.

**Table 6:** Cataract surgical coverage according to VA and sex of individuals aged ≥50 years in Costa Rica (2015).

	Men	Women	Total
***Cataract surgical coverage (eyes)***			
VA < 3/60	70.0	78.2	74.4
VA < 6/60	58.4	67.5	63.2
VA < 6/18	40.2	45.1	42.8
***Cataract surgical coverage (individuals)***			
VA < 3/60	86.1	90.9	88.9
VA < 6/60	70.5	81.2	76.6
VA < 6/18	56.5	63.1	60.3

VA: visual acuity

The main reason why cataract surgery was not performed for individuals with a BCVA of ≤6/60 was “need not felt” (48%), followed by “fear” (14.6%) and “not aware that treatment is possible” (12.2%). The remaining subjects had been evaluated by an ophthalmologist and were not considered eligible for surgery (8.5%), were awaiting surgery (6.1%), or had problems with their insurance policies (4.9%).

A “good” or “very good” visual outcome (PVA, ≥6/18) after cataract surgery was observed for 57.1% of eyes; this proportion rose to 64.6% if postsurgical refractive errors were corrected (VA was retested with a pinhole; Table 7).

**Table 7:** PVA and BCVA after cataract surgery in individuals aged ≥50 years in Costa Rica (2015).

	PVA		BCVA	
	Eyes	%	n	%
***Very good*: *can see*, *6/12***	119	36.0%	162	48.9%
***Good*: *can see*, *6/18***	70	21.1%	52	15.7%
***Moderate*: *can see*, *6/60***	80	24.2%	63	19.0%
***Poor*: *cannot see*, *6/60***	62	18.7%	54	16.3%
**Total**	**331**	**100%**	**331**	**100%**

PVA: presenting visual acuity, BCVA: best-corrected visual acuity

There were minimal differences in the outcomes of surgery according to the time (after onset; Table 8) and place (Table 9) of surgery (Tables 8 and 9).

**Table 8:** Outcomes of cataract surgery (PVA) according to the time of surgery (after onset) in individuals aged ≥50 years in Costa Rica (2015).

				3 years after onset		4–6 years after onset		≥7 years after onset		Total	
			
				Eyes	%	Eyes	%	Eyes	%	Eyes	%
***Very good*: *can see*, *6/12***				52	38.0%	30	41.1%	37	30.6%	**119**	**36.0%**
***Good*: *can see*, *6/18***				32	23.4%	14	19.2%	24	19.8%	**70**	**21.1%**
***Moderate*: *can see*, *6/60***				31	22.6%	17	23.3%	32	26.4%	**80**	**24.2%**
***Poor*: *cannot see*, *6/60***				22	16.1%	12	16.4%	28	23.1%	**62**	**18.7%**
**Total**				**137**	**100.0%**	**73**	**100.0%**	**121**	**100.0%**	**331**	**100.0%**

PVA: presenting visual acuity

**Table 9:** Outcomes of cataract surgery (PVA) according to the place of surgery in individuals aged ≥50 years in Costa Rica (2015).

	Public hospital		Charitable hospital		Private Hospital		Ocular health camp		Total	

	Eyes	%	Eyes	%	Eyes	%	Eyes	%	Eyes	%
***Very good*: *can see*, *6/12***	80	33.8	11	36.7	28	44.4	0	0.0	**119**	**36.0**
***Good*: *can see*, *6/18***	50	21.1	6	20.0	14	22.2	0	0.0	**70**	**21.1**
***Moderate*: *can see*, *6/60***	60	25.3	7	23.3	13	20.6	0	0.0	**80**	**24.2**
***Poor*: *cannot see*, *6/60***	47	19.8	6	20.0	8	12.7	1	100.0	**62**	**18.7**
**Total**	**237**	**100.0**	**30**	**100.0**	**63**	**100.0**	**1**	**100.0**	**331**	**100.0**

PVA: presenting visual acuity

Our results also revealed that the largest number of eyes that had undergone cataract surgery belonged to men and women aged ≥70 years (Table 10).

**Table 10:** Distribution of eyes (individuals aged ≥50 years) in Costa Rica (2015) according to the sex and age of the patient at the time of cataract surgery.

	Men		Women		Total	

Age (years)	Eyes	%	Eyes	%	Eyes	%
1–29	1	0.8%	1	0.5%	**2**	**0.6%**
30–39	0	0.0%	1	0.5%	**1**	**0.3%**
40–49	10	7.8%	14	6.9%	**24**	**7.3%**
50–59	7	5.4%	29	14.4%	**36**	**10.9%**
60–69	40	31.0%	52	25.7%	**92**	**27.8%**
70–79	43	33.3%	68	33.7%	**111**	**33.5%**
80+	28	21.7%	37	18.3%	**65**	**19.6%**
**Total**	**129**	**100.0%**	**202**	**100.0%**	**331**	**100.0%**

### Diabetic retinopathy

In total, 553 of the 2,493 subjects exhibited diabetes (estimated prevalence, 22.2%; 95% CI: 20.4%–24.0%). The prevalence was significantly higher among women (22.4%; 95% CI: 22.1%–26.7%) than among men (18.4%; 95% CI: 15.9%–21.0%). Of the total, 93.9% subjects were aware of their condition and 6.1% were diagnosed for the first time. Among the known diabetics, 30.1% exhibited an RBG level of ≥200 mg/dl, 51.6% had not undergone previous screening for diabetic retinopathy, and 23.3% had received their last examination >2 years ago. Only 19.3% of subjects had been screened for diabetic retinopathy within the last year.

Diabetic retinopathy accounted for 6.3% cases of blindness, 2% cases of severe visual impairment, 2.8% cases of moderate visual impairment, and 0.5% cases of early visual impairment. A total of 21.6% (95% CI: 18.0%–25.2%) subjects had diabetic retinopathy of any grade (Table 11; using the Scottish Diabetic Retinopathy Grading Scheme [9]), with 6.2% (95% CI: 3.8%–8.7%) exhibiting sight-threatening diabetic retinopathy. The latter group of patients would require expensive treatments performed by trained specialists in well-equipped facilities, such as posterior vitrectomy performed by a retinal surgeon.

**Table 11:** Prevalence of DR among individuals aged ≥50 years in Costa Rica (2015).

	n	Individuals with diabetes % (95% CI)
No DR (R0)	399	77.6 (73.9–81.3)
Background DR: mild (R1)	72	14.0 (10.9–17.1)
Background DR: observable (R2)	12	2.3 (1.2–3.5)
Background DR: referable (R3)	18	3.5 (1.9–5.1)
Proliferative DR (R4)	9	1.8 (0.5–3.0)
Ungradable	4	0.8 (0.0–1.5)
**Any retinopathy**	111	21.6 (18.0–25.2)
No maculopathy (M0)	444	86.4 (83.3–89.4)
Maculopathy: observable (M1)	29	5.6 (3.5–7.7)
Maculopathy: referable (M2)	27	5.3 (3.1–7.4)
Ungradable (M6)	4	0.8 (0.0–1.5)
**Any maculopathy**	56	10.9 (7.9–13.8)
**Any retinopathy and/or maculopathy**	121	23.5 (20.0–27.1)
**Sight-threatening DR (R4 and/or M2)**	32	6.2 (3.8–8.7)
**Any laser scars**	24	4.7 (2.7–6.6)

DR: diabetic retinopathy

## Discussion

In the present study, we estimated the prevalence and causes of avoidable blindness and visual impairment and the prevalence of diabetic retinopathy in individuals aged ≥50 years in Costa Rica.

The prevalence of blindness in Costa Rica (1.7%; 95% CI: 1.2%–2.2%) was found to be the lowest when compared with Central America (2.1%; 95% CI: 1.7%–2.7%), Panama (3.0%; 95% CI: 2.3%–3.6%), El Salvador (2.4%; 95% CI: 2.2–2.6), and Honduras (1.9%) [10]. Moreover, our results showed no difference in the prevalences of blindness and visual impairment between men and women, unlike the results of a recent meta-analysis [11]. In the year 2010, the estimated prevalence of blindness among men and women aged ≥50 years in Central America was 1.8% (95% CI: 1.4%–2.4%) and 2.0% (95% CI: 2.0%–3.1%), respectively, while that of moderate to severe visual impairment was 9.8% (95% CI: 7.6–12.6) for men and 11.4% (95% CI: 8.7%–14.7%) for women [10]. The data obtained for Costa Rica are within the expected range for Central American countries.

The prevalence of blindness caused by untreated cataract was within the expected range for Costa Rica [12]. Moreover, the prevalence of severe visual impairment due to untreated cataract (66.7%) was comparable with that in Honduras (60.7%) [12] and Panama (69.2%) [13]. On the other hand, the prevalence of moderate visual impairment due to untreated cataract was much higher in Costa Rica than in other countries because uncorrected refractive errors, which are typically the main cause of moderate visual impairment, are reduced due to the increased coverage and access to optical correction measures. Further research is needed to clarify this result.

We can hypothesize that individuals with cataract usually do not experience visual impairment in the early stages, because cataract develops gradually and becomes significant at a point when the activities and visual needs of those affected have diminished. Nevertheless, if we are trying to eradicate cataract as a cause of blindness and achieve better visual outcomes, we need to establish health policies designed to overcome the most common barriers against cataract surgery through health education and improved management of ophthalmic services, considering that poor patient selection was also a factor for poor visual outcomes after surgery (55.6%). Clinicians should be aware that individuals operated at a more advanced age exhibit a greater probability of developing comorbidities and complications during or after surgery.

Laser therapy for diabetic retinopathy can prevent severe retinal damage if it is promptly performed when required, and the decision for this treatment has to be made by an ophthalmologist. However, an ophthalmologist cannot screen all individuals with diabetes because of the lack of the required equipment and high costs. For example, for an estimated 235,000 individuals with diabetes aged ≥50 years in Costa Rica, 35 full-time ophthalmologists would be required to perform annual screenings in accordance with official guidelines. Therefore, only individuals with early diabetic retinopathy should be referred for specialized examinations, and interpretation of fundus photographs acquired with mydriatic or nonmydriatic cameras by trained professionals serves as a certain and cost-effective screening method for patients [14]. Conventional screening using direct ophthalmoscopy performed by general practitioners has proven to be ineffective because of its low sensitivity [15].

The strength of this study is that it adopted an accepted methodology that allows for comparison with studies performed in other countries, and future follow–up studies can use this methodology to evaluate the impact or lack of action taken.

This study also has some limitations. First, the subject selection procedure was such that more sensitive diabetes screening tools other than RBG estimation in a capillary blood sample could not be used. Second, the low response rate could be a possible and an important source of bias. Only 76% of eligible subjects were examined. This could have led to overestimation of the prevalences of visual impairment and diabetic retinopathy [11].

## Conclusions

In conclusion, avoidable blindness accounts for 68.8% cases of blindness in Costa Rica. The main cause is untreated cataract; therefore, strategies should be directed toward overcoming barriers against cataract surgery, increasing CSC, and improving the surgical outcomes. Accordingly, a development plan for ophthalmic services is required, which should motivate general practitioners to comprehensively evaluate patients with ophthalmological pathologies that do not strictly require evaluation by specialists; this will allow the maximum usage of all existing resources so that the actual needs of the population with risk factors for blindness and visual impairment are met. Moreover, establishment of protocols for the allocation of resources is necessary to ensure timely intervention for the more common pathologies. Even then, the number of ophthalmologists in training every year needs to increase in Costa Rica, where the requirement for cataract surgeries is too high and the number of specialist clinics too low. Moreover, the prevalence of diabetes was significantly different between men and women, and further studies regarding this aspect are required before the development of relevant health policies and strategies. Diabetic retinopathy screening should be improved with the implementation of proven methods such as the use of mydriatic cameras operated by trained personnel for the identification of any risk factors warranting prompt referral to an ophthalmologist [14]. Regional screening for glaucoma has not proven to be cost-effective for large populations; however, areas with an expected high prevalence associated with the presence of known risk factors, such as ethnicity (Caribbean population), age (>40 years), and a family history of diabetes or glaucoma [12], could require such regional screening programs. After the screening survey, the results of which have been presented in this article, Costa Rica has already begun the process of developing an Eye Healthcare National Program, which adopts specific policies, strategies, tactics, and operational objectives to face the challenge of providing efficient and timely ocular health care to the population. In addition, our results are expected to guide new efforts toward the treatment and prevention of avoidable blindness.

## References

[1] World Health Organization. Prevention of Blindness and Visual Impairment. Fact sheet Global Data 2010. 2013 Available from: http://www.who.int/blindness/data_maps/VIFACTSHEETGLODAT2010full.pdf?ua=1

[2] World Health Organization. Programmes. Prevention of Blindness and Visual Impairment. 2014 Available from: http://www.who.int/blindness/AP2014_19_Spanish.pdf?ua=1

[3] Vision 20/20. Vision 20/20 Boletín Trimestral. 2015. Available from: https://vision2020la.wordpress.com/2014/07/14/situacion-de-la-cirugia-de-catarata-en-america-latina

[4] Resnikoff S, Felch W, Gauthier TM, Spivey B. The number of ophthalmologists in practice and training worldwide: a growing gap despite more than 200 000 practitioners. Br J Ophthalmol. 2012;96: 783–787. 10.1136/bjophthalmol-2011-301378 22452836

[5] Kuper H, Polack S, Limburg H. Rapid assessment of avoidable blindness. Community Eye Health. 2006;19: 68–69. 17515970 PMC1871676

[6] Limburg H. Rapid Assessment of Avoidable Blindness. Community Eye Health Journal. 20 12 2013. [On line]. Available: Community Eye Health. PMC1871676 17515970

[7] Limburg H, Barria von-Bischhoffshausen F, Gomez P, Silva JC, Foster A. Review of recent surveys on blindness and visual impairment in Latin America. Br J Ophthalmol. 2008;92: 315–339. 10.1136/bjo.2007.125906 18211928

[8] Barcelo A, Gregg E, Perez-Flores E, Wong R, Gerzoff R, Cafiero E, et al Survery of Diabetes, Hypertension, and Chronic Disease Risk Factors Belice, San Jose, San Salvador, Ciudad Guatemala, Managua and Tegucigalpa. 2009: PAHO Libraries, Washington D.C.

[9] Zachariah S, Wykes W, Yortson D. Grading diabetic retinopathy (DR) using the Scotish grading protocol. Community Eye Health. 2015;28: 72–73. 27418727 PMC4944099

[10] Silva JC. Una evaluación comparativa de la ceguera y la deficiencia visual evitables en siete países latinoamericanos, prevalencia cobertura y desigualdade. Revista Panamericana de Salud Pública. 2015;37: 21–22.

[11] Flaxman SR, Bourne RRA, Resnikoff S, Ackland P, Braithwaite T, Cicinelli MV, et al Global causes of blindness and distance vision impairment 1990–2020: a systematic review and meta-analysis. Lancet Glob Health. 2017;5: 1221–1234. 10.1016/S2214-109X(17)30393-5 29032195

[12] Alvarado D, Rivera B, Lagos L, Ochoa M. Encuesta nacional de ceguera y deficiencia visual evitables en Honduras. Rev Panam Salud Pública. 2014;36: 300–305. 25604099

[13] Lopez M, Brea I, Yee R. Encuesta de ceguera y deficiencia visual evitable en Panamá. Rev Panam Salud Public. 2014;36: 355–360. 25711745

[14] American Diabetes Association. Diabetic Retinopathy. Diabetes Care. 2002;25: S90–S93.

[15] Williams G, Scott I, Haller J, Maguire A, Marcus D, McDonald H. Single-field fundus photography for diabetic retinopathy screening: a report by the American Academy of Ophthalmology. Ophthalmology. 2004;111: 1055–1062. 10.1016/j.ophtha.2004.02.004 15121388

